# Search templates that incorporate within-face variation improve visual search for faces

**DOI:** 10.1186/s41235-018-0128-1

**Published:** 2018-09-26

**Authors:** James D. Dunn, Richard I. Kemp, David White

**Affiliations:** School of Psychology, UNSW Sydney, Sydney, NSW 2052 Australia

**Keywords:** Face recognition, Visual search, Search template, Within-face variability, Familiarity, Face-in-a-crowd

## Abstract

Searching for unfamiliar faces in crowds is an important task in modern society. In surveillance and security settings, it is sometimes critical to locate a target individual quickly and accurately. In this study, we examine whether we can improve search efficiency in these visual search tasks by changing the face information that is provided to participants. In Experiment 1, we compare speed and accuracy of visual search when searching for unfamiliar and familiar faces after being exposed to either a single exemplar image or a face average created from multiple images of the target face. In Experiment 2, we compare search efficiency when single exemplars and multiple exemplars are provided. Consistent with studies of unfamiliar face matching tasks, we find that, relative to a single image, having multiple images of the target improves the accuracy of visual search. In Experiment 3, we compared search performance for face averages and multiple exemplars while also varying crowd size. Multiple exemplars conferred an additional advantage over face averages, suggesting that exposure to within-face variability results in the best search performance. We discuss the implications of these findings for face-in-a-crowd search and visual search tasks more generally.

**Electronic supplementary material:**

The online version of this article (10.1186/s41235-018-0128-1) contains supplementary material, which is available to authorized users.

## Significance statement

Finding persons of interest in crowded scenes is an increasingly important task for maintaining public safety. However, studies examining how well people perform this task have used studio-quality images that do not reflect the diverse and challenging viewing conditions encountered in unconstrained environments. Here, we test people’s ability to find unfamiliar faces in crowds using images that show the day-to-day variations in appearance that are typically encountered in real-world tasks. We find that people make many errors when searching for unfamiliar faces in crowds, selecting the wrong individual on over half of decisions. Performance was far better for familiar faces and when participants were exposed to either a face average or multiple images of an unfamiliar face before the search task. These results suggest that methods for optimizing face learning can provide substantial benefits to accuracy and efficiency of visual search for faces in unconstrained environments.

## Background

Recently, the identification of people in crowds has emerged as an important task in both crime prevention and law enforcement. To pre-empt potential threats, police attempt to identify whether known antagonists are present at large public gatherings (Dodd, 2017). Alternatively, police can review footage captured on closed-circuit television (CCTV) to locate and identify suspects at large-scale public events after an incident has occurred (BBC, 2011). In both scenarios, success can rely on the identification of suspects from their faces when they are present within the crowd as it is an important cue for person identification (Burton, Wilson, Cowan, & Bruce, 1999). While these decisions can be made with the assistance of automated face recognition software, because this technology is currently unreliable when used to identify suspects in crowds (Evison, 2018; Lamb, 2017; Stacy, 2017), a human operator will often have to make the final identification decision (White, Dunn, Schmid, & Kemp, 2015). Therefore, it is important to consider the human factors that may limit the effectiveness of face-in-the-crowd identification.

Although there have been a few studies investigating how well people search for faces, these studies may not capture the full difficulty of visual search in real-world conditions. Participants of these tasks are typically shown high-quality images of faces, captured in ideal studio conditions (di Oleggio Castello, Wheeler, Cipolli, & Gobbini, 2017; Ito & Sakurai, 2014; Tong & Nakayama, 1999). This may explain why accuracy on these tasks was both high, with no more than 1.3% errors, and fast, with targets being located on average within 2 s (di Oleggio Castello et al., 2017; Ito & Sakurai, 2014; Tong & Nakayama, 1999). However, when searching for faces in the real world, the appearance of a face can vary widely. For example, a face can be subject to variability in viewpoint, expression, and lighting which are all detrimental for recognizing an unfamiliar face (for review, see Johnston & Edmonds, 2009). Likewise, searches using CCTV footage typically occur in ambient environments where images with poor lighting, large distances from the camera, and low resolutions are common (Introna & Nissenbaum, 2009). When faced with these challenging conditions, it is likely that visual search performance will decline.

In a recent demonstration of real-world face-search performance by experts, Davis, Forrest, Treml, and Jansari (2018) recruited two groups of police officers from the London Metropolitan Police Service. These participants completed a naturalistic task that assessed their ability to identify suspects in CCTV footage. One group were seven current and former members of the Super-Recogniser Unit, who regularly perform various identification tasks as part of their work and have shown higher accuracy than controls on tests of unfamiliar face matching (Robertson, Noyes, Dowsett, Jenkins, & Burton, 2016). The other group served as a control sample and were police officers who do not specialize in face identification. Worryingly, they found that both groups had a high proportion of errors. When attempting to identify the eight target people within the 18 min of footage, officers from the Super-Recogniser unit wrongly identified 2.3 of “innocent bystanders” present in the video and missed roughly one-fifth (19%) of their “suspects.” Accuracy for the Control participants was lower than for members of the Super-Recogniser unit, as Controls selected, on average, 4.1 innocent bystanders and missed 29% of their suspects. Nevertheless, both groups performed poorly on this task, illustrating just how challenging visual search tasks can be in applied settings, even for professional staff who do this as part of their daily work.

However, recent research suggests that familiarity could be important for overcoming these challenges. In one of a handful of papers that has examined visual search performance for faces, Tong and Nakayama (1999) found that familiar faces were located more quickly than unfamiliar faces from among distractor faces. They argued that these differences in performance might be due to a more robust memory representation, which facilitates faster and more accurate search. This finding is consistent with the transformative effect that familiarity has on performance across a variety of face identification tasks. For example, when deciding if two images of faces are of the same person or two different people, viewers have great difficulty when faces are unfamiliar, but experience no such difficulty when faces are familiar (Burton et al., 1999; Hancock, Bruce, & Burton, 2000; White, Burton, Jenkins, & Kemp, 2014).

An important benefit of familiarity is how it enables recognition despite considerable variability in appearance. For example, Jenkins, White, Van Montfort, and Burton (2011) asked participants to sort 40 face photographs by identity, so that any images of the same person would appear in the same pile. Unknown to participants, these 40 photographs only depicted two individuals. However, because the face in the photographs varied naturally in appearance, participants unfamiliar with the identities sorted images into on average seven different piles. Conversely, participants familiar with the identities invariably found the correct solution, sorting the images into two piles. This suggests that becoming familiar with a face enables people to cope with the substantial within-face variability that is encountered because of day-to-day changes in appearance.

Following this work, one promising way to improve real-world visual search performance for unfamiliar faces is to familiarize participants with targets before search. Here, we explore two alternatives to using single images as visual search targets that may compensate for viewer’s lack of familiarity with a face. First, we examine whether providing an average image of the target, created by statistically aggregating several different images of the target person, is able to improve search performance. It has been suggested that averaging reduces the contribution of aspects that vary across different images—such as lighting, pose, or expression—while emphasizing the features that are consistent across images (Burton, Jenkins, Hancock, & White, 2005), thereby improving the signal-to-noise ratio of identifying information in the image. This method has been shown to improve both human and automated familiar face recognition (Burton et al., 2005; Jenkins & Burton, 2008) and unfamiliar face matching accuracy (White et al., 2014).

Second, we examine whether providing multiple images of the target’s face can improve search performance. Recent studies have shown that providing multiple images improves the accuracy of simultaneous face matching (Bindemann & Sandford, 2011; Menon, White, & Kemp, 2015b; White et al., 2014), sequential face matching (Menon, White, & Kemp, 2015a), and recognition memory (Etchells, Brooks, & Johnston, 2017; Murphy, Ipser, Gaigg, & Cook, 2015). The key difference between this approach and providing image averages is that by presenting multiple images, the viewer experiences the way in which a face varies between images. Exposure to this within-face variability is believed to enhance the construction of abstractive representations, enabling generalization to novel instances for a face (Etchells et al., 2017; Menon et al., 2015a; Murphy et al., 2015; Ritchie & Burton, 2017; White et al., 2014) and so may also enable better search performance when images are unconstrained.

**Fig. 1:**
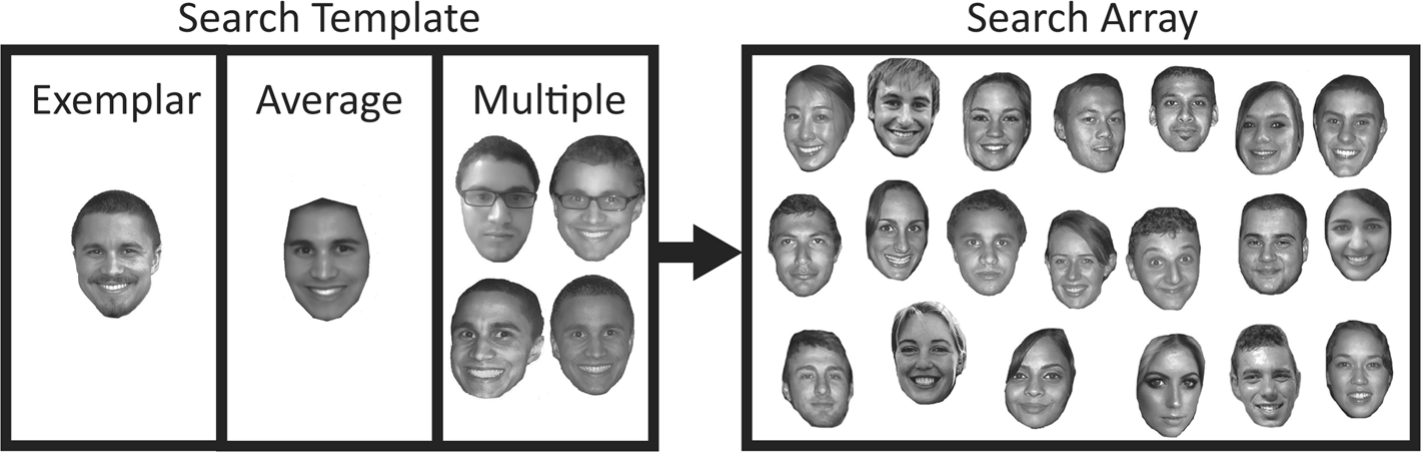
Example showing the same target face with the search templates and search arrays used in all experiments. On each trial one of three search templates (*left*) was shown on screen before the search array (*right*). Participants had to find the target in the array by clicking on the faces in the image, which in the example is located in the second row, third from the left. In Experiment 1, we compare search performance when participants were presented with a single exemplar image vs an average image. In Experiment 2, we compare performance in single vs multiple exemplar conditions and in Experiment 3 we compare average vs multiple images. Images are representative of the materials used in tasks, but for copyright restrictions, we are not able to provide the actual materials used in our studies

Across three experiments, we test the accuracy and speed with which participants find a novel image of a target identity among multiple distractor identities. Studies of visual search behavior for faces have used images that are standardized in terms of lighting, expression, and pose; so, earlier experiments do not provide an indication of the accuracy of search in real-world environments. Our aim is to develop methods that can be used to produce robust improvements in real-world tasks. Therefore, we use “ambient” images that have natural day-to-day variations in appearance from one image to the next, such that the appearance of a person pictured in a target image can vary markedly from their appearance in the search array (see Fig. 1). Further, previous studies of visual search for faces have used search arrays that were relatively small, consisting of only four (Ito & Sakurai, 2014), six (Tong & Nakayama, 1999), or eight images (Mestry, Menneer, Cave, Godwin, & Donnelly, 2017). In many important real-world tasks, people have to find a target face within crowds containing hundreds or even thousands of bystanders; therefore, in a final experiment, we examine how visual search performance varies as a function of array size.

## Experiment 1

In Experiment 1, we provide the first test of visual search performance for familiar and unfamiliar faces, when images are sampled “in the wild” from relatively unconstrained environments (see Fig. 1). In addition, we examine whether we can improve search efficiency by manipulating the content of the “search template,” i.e. the target image shown to participants before search (Hout & Goldinger, 2015; Olivers, Peters, Houtkamp, & Roelfsema, 2011). Participants are either shown a single image of the target—as is standard in the visual search paradigm—or are shown an average face that is generated by aggregating information across multiple images of the target face (Fig. 1, left). Based on earlier work showing that average images improve the accuracy of face identification decisions (Burton et al., 2005; White et al., 2014), we predict that average images will improve visual search performance relative to the single image condition.

### Method

#### Participants

Twenty-six undergraduate students (19 women, mean age 18.7 years, SD = 0.9) took part in the experiment in exchange for course credit. All participants reported normal or corrected-to-normal visual acuity and normal color vision. Informed consent was obtained before the experiment.

#### Materials and procedure

Exemplar images were taken from a dataset containing 19 images each of 20 local Dutch celebrities (unfamiliar) and 20 famous international celebrities (familiar). These images were obtained via Google Image Search. Selected images were cropped to remove the background and changed into grayscale. The resolution of each of the images was 190 × 285 pixels. Selected images showed the full face, with no spectacles present, but otherwise were sampled to vary naturally in image level qualities like lighting, expression, pose, and head angle. Averages were created by statistically averaging the 19 exemplars of each identity. This process required anatomical landmarks to be placed on each image to map the facial features. We then used the average RGB values for each pixel in a linear space to generate a “shape-free” texture map of the face. The texture information was then morphed onto the average shape, calculated from the landmarks placed on the original images, to produce the average image for each identity (Burton et al., 2005; White et al., 2014). We normalized average and exemplar images to match average luminance profiles using the SHINE toolbox (Willenbockel et al., 2010). Examples of exemplar and average images are shown in Fig. 1.

Participants were tested individually and the experiment was implemented in Psychtoolbox for MATLAB (Brainard, 1997). Participants completed 80 trials of a visual search task that required them to find a target identity within a display. Each of the 20 Dutch and international celebrities were shown twice in this experiment, once in each condition. Each trial involved three stages. First, participants performed a familiarity check that asked them to either type the name or describe why they knew the celebrity shown on screen.

A celebrity was only considered as being familiar to a participant if they were either able to correctly name the celebrity or give an accurate description of why they remembered that person.^1^ For example, for Tom Cruise we coded the following responses by participants as indicating familiarity: “Tom Cruise,” “From Mission Impossible,” or “Jumped on Oprah’s couch;” while incorrect statements such as “Brad” or general descriptions such as “American actor” were not considered specific enough and were therefore labelled as unfamiliar for analysis. Although each identity was presented twice in the experiment, once with an exemplar and once with the average, participants only had to recognize a celebrity once to be coded as being familiar. Using the described criteria for familiarity, participants correctly identified on average 11 of the 20 international celebrities (SD = 4). As anticipated, none of the 20 Dutch celebrities were familiar to our participants.

On the second screen in a trial, participants were first instructed to click the center of the screen in order to localize the position of the mouse pointer. They were then shown the same image of the search template along with the text “Find this person” for 3 s. For the face average condition, this screen showed the face average for the target identity. For the exemplar condition, a random image of that identity was shown, which differed for each participant. Finally, on the last screen, participants were shown a search array consisting of 20 images, where one image was always of the target and the remaining 19 were distractor images of other identities (Fig. 1). Participants had to respond by using the mouse to click on the image they believed to be the target and they were instructed to do so as quickly and accurately as possible. The position of the target within the search array was always randomized and all images remained on screen until a decision was made. The target image was always a different image to that shown during the familiarity check and target preview screens. The identity of the distractors in the search array was dependent on the familiarity of the target, with international celebrities being used as distractors for familiar targets and Dutch celebrities being used as distractors for unfamiliar targets. Accuracy was calculated as the percentage of correct selections made in each condition.

### Results

**Fig. 2:**
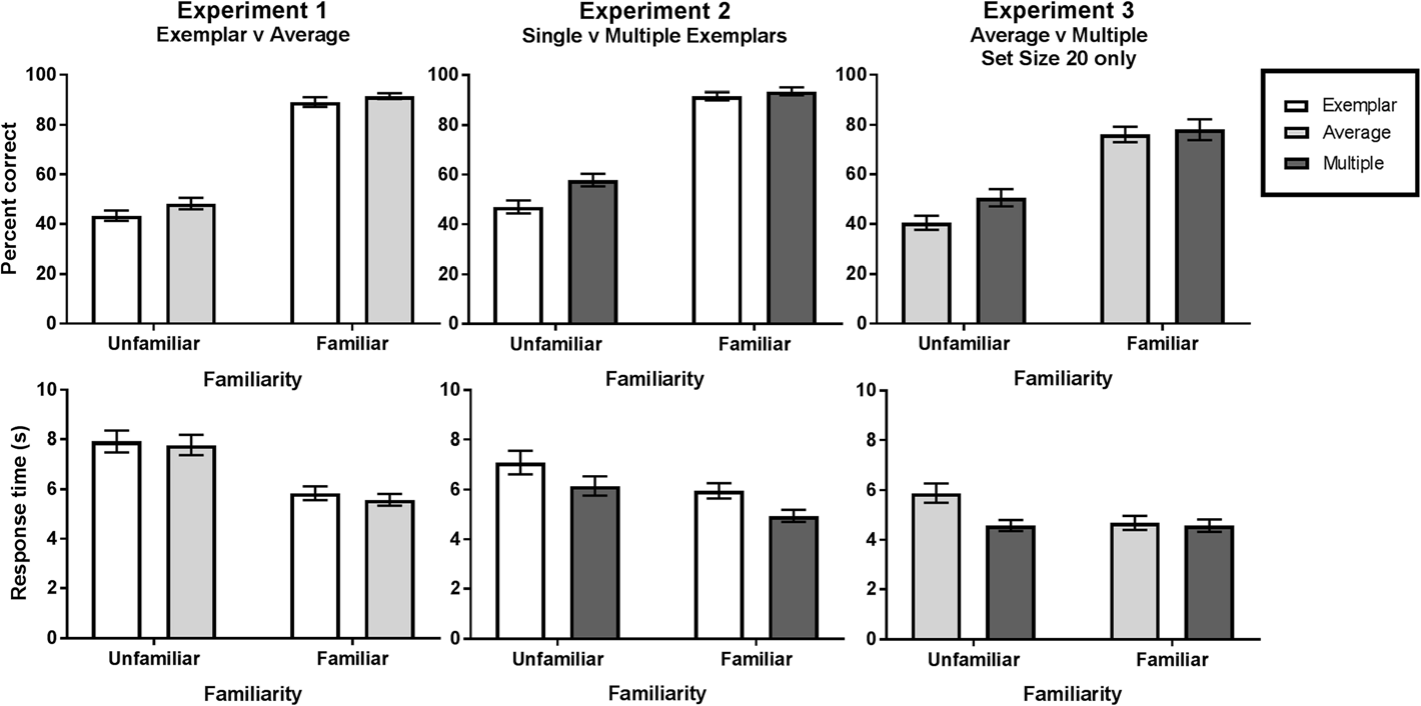
Mean accuracy and response time for each experiment. To add comparison between experiments, the results for Experiment 3 only include the Set Size 20 condition. *Error bars* denote ±1 standard error

Two 2 × 2 repeated measures ANOVAs were used to analyze accuracy and response time separately, with Familiarity (unfamiliar, familiar) and Template (exemplar, average) as the within-participants factors (Fig. 2). Because we were only interested in the speed of correct decisions, response time data from incorrect trials were not analyzed. For the response time analysis, we also disregarded any trial with a premature response (< 150 ms) or that was excessively long (> 3 SDs above that participant’s overall mean response time).

#### Accuracy

For accuracy, there was a significant main effect of Familiarity, *F*(1, 25) = 482.74, *p* < 0.001, η_p_^2^ = 0.951, with familiar faces being correctly located on more trials than unfamiliar faces (familiar = 90.4%, unfamiliar, 45.8%). There was also a main effect for Template, *F*(1, 25) = 5.52, *p* = 0.027, η_p_^2^ = 0.181, with participants more accurately selecting the face after seeing a face average template than after seeing a random exemplar template (average = 70%, exemplar = 66.3%). There was no significant interaction between factors, *F*(1, 25) = 0.57, *p* = 0.457, η_p_^2^ = 0.022.

#### Response time

For response time, there was a significant main effect of Familiarity, *F*(1, 25) = 52.48, *p* < 0.001, η_p_^2^ = 0.677, with familiar faces being correctly found faster than unfamiliar faces (familiar = 5.7 s, unfamiliar = 7.8 s). There was no significant main effect for Template, *F*(1, 25) = 1.15, *p* = 0.293, η_p_^2^ = 0.044, and no significant interaction between factors, *F*(1, 25) = 0.17, *p* = 0.682, η_p_^2^ = 0.007.

### Discussion

The results of Experiment 1 demonstrate the discrepancy between search performance for unfamiliar and familiar faces. With unfamiliar faces, performance was highly error prone, as participants selected the wrong face more often then they selected the correct one. Conversely, familiar faces were found faster and more accurately than unfamiliar faces. In fact, participants only made errors on 10% of trials when searching for a familiar face, showing comparable error rates to previous tests of familiar face matching that used unconstrained images (for example, White et al., 2014). However, we are also encouraged by these results, as search performance was improved by using face averages. Specifically, we found improvements in search accuracy when participants saw an average face image of the target before search compared to a randomly selected exemplar. This extends results showing that averages improve accuracy in unfamiliar face matching tasks where images are presented simultaneously on the screen (White et al., 2014). These results suggest that images that provide a more robust representation of the target can improve search performance for faces.

These results suggest that providing face averages to people that are searching for faces in crowds, for example security and police personnel, may result in better search accuracy. However, because face averages require multiple images to construct them, it may be more beneficial to give multiple images to officers in the field. Indeed, there may be additional benefits of providing multiple images for face search, as recent work has found that exposure to within-face variability presented across multiple images benefits face matching accuracy (Bindemann & Sandford, 2011; Etchells et al., 2017; Menon et al., 2015a, 2015b; White et al., 2014). In the next experiment, we simulate exposure to variability by providing a search template consisting of multiple images of the target to determine whether this also improves search performance.

## Experiment 2

In this experiment, we measured whether multiple images of a target would lead to a more robust search template and improve search performance relative to one image. We again asked participants to find a target that was either an unfamiliar or a familiar face after showing them either one or four images of that face.

### Method

#### Participants

Twenty-six undergraduate students (19 women, mean age 19.0 years, SD = 1.5) took part in the experiment in exchange for course credit. All participants reported normal or corrected-to-normal visual acuity and normal color vision. Informed consent was obtained before the experiment.

#### Materials and procedure

Materials and procedure were identical to Experiment 1, except that the average image condition was replaced by a four-exemplar condition in which four exemplars were randomly selected from the set of 19 images of each identity and presented simultaneously as the search template (see Fig. 1). The four images selected were different for each participant. Using the familiarity criteria from Experiment 1, an average of 14 of the 20 international celebrities were correctly identified and thus confirmed to be familiar (SD = 3). None of the Dutch celebrities were familiar.

### Results

As in the previous experiment, two 2 × 2 repeated measures ANOVAs were used to analyze accuracy and response time separately, with Familiarity (unfamiliar, familiar) and Template (single image, multiple images) as the factors (Fig. 2).

#### Accuracy

For accuracy, there was a significant main effect of Familiarity, *F*(1, 25) = 356.82, *p* < 0.001, η_p_^2^ = 0.935, with familiar faces found more accurately than unfamiliar faces (familiar = 92.6%, unfamiliar = 52.4%). The main effect of Template was also significant, *F*(1, 25) = 14.56, *p* = 0.001, η_p_^2^ = 0.368, with the multiple image templates having higher accuracy than single image templates (multiple = 75.7%, single = 69.3%). There was a significant interaction between these factors, *F*(1, 25) = 11.08, *p* = 0.003, η_p_^2^ = 0.307. Simple main effects analysis reveal that participants were more accurate in the multiple image condition when searching for an unfamiliar face, *F*(1, 25) = 17.68, *p* < 0.001, η_p_^2^ = 0.414, but this difference was not significant for familiar faces, *F*(1, 25) = 1.52, *p* = 0.229, η_p_^2^ = 0.057.

#### Response time

For response time, there was a significant main effect of Familiarity, *F*(1, 25) = 15.68, *p* = 0.001, η_p_^2^ = 0.154, with familiar faces being correctly found faster than unfamiliar faces (familiar = 5.4 s, unfamiliar = 6.6 s). There was also a significant main effect for Template, *F*(1, 25) = 31.38, *p* = 0.001, η_p_^2^ = 0.557, with the target being found faster after a multiple image template than a single image template (multiple = 5.5 s, single = 6.5 s). There was no significant interaction between factors, *F*(1, 25) = 0.08, *p* = 0.776, η_p_^2^ = 0.003.

### Discussion

This study replicated the large differences in search speed and accuracy between unfamiliar and familiar faces found in Experiment 1, with familiar faces found more quickly and accurately than unfamiliar faces. Furthermore, showing multiple images of a target resulted in better search performance, with targets found both more quickly and more accurately with four images compared to one. This suggests that the search templates elicited from multiple images are more robust than the template based on a single image. This is consistent with the benefit of multiple images observed in studies of unfamiliar face matching (Bindemann & Sandford, 2011; Menon et al., 2015a, 2015b; White et al., 2014). Importantly for face search, multiple images showed the largest improvement for unfamiliar faces, suggesting that this may be a practical way to enhance search performance in applied settings.

Multiple images have an added benefit over face averages in that they provide information about how a facial appearance varies from one encounter to the next. In real-world tasks where the appearance of the target face is difficult to predict, having some information about the possible range of appearances may be particularly beneficial. In the next experiment, we test whether exposure to variability in appearance improves search accuracy relative to exposure to modal appearance, by comparing search performance when face averages and multiple images are provided as search templates.

## Experiment 3

In Experiment 3, we compare search performance for face average and multiple images search templates. We also investigate the impact of set size on performance, by varying the number of distractors in the array. This enables us to estimate the relationship between the set size and performance, which is an important consideration when estimating the accuracy of visual search in real-world settings. By estimating this “search-slope” function, it is possible to estimate how speed and accuracy would be impacted by larger crowd sizes (Tong & Nakayama, 1999; Treisman & Souther, 1985).

### Method

#### Participants

Twenty-six undergraduate students (17 women, mean age 19.2 years, SD = 1.9) took part in the experiment in exchange for course credit. All participants reported normal (or correct-to-normal) visual acuity and normal color vision. Informed consent was obtained before the experiment.

#### Materials and procedure

Experiment 3 employed the same materials and procedure used in Experiments 1 and 2 except that we varied the search Set Size (either 5, 10, or 20 images). Each target identity appeared three times (once at each set size), randomly located in the array along with 4, 9, or 19 fillers. The assignment of search templates to set size conditions was randomized across stimulus identities and counterbalanced across participants. In this experiment, an average of 13 of the 20 international celebrities (SD = 4) were correctly identified. None of the Dutch celebrities were familiar to any of the participants.

### Results

**Fig. 3:**
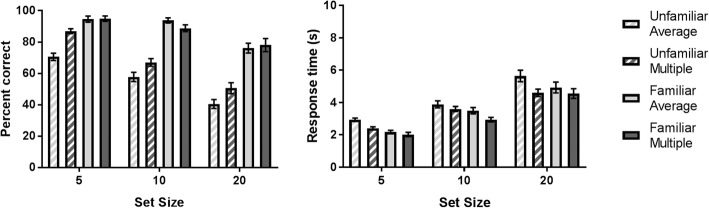
Mean accuracy and response time for Experiment 3 by Set Size. *Error bars* denote ±1 standard error

Two 2 × 2 × 3 repeated measures ANOVAs were used to analyze the accuracy and response time of participant’s responses with Familiarity (unfamiliar, familiar), Template (average, multiple), and Set Size (5, 10, 20) as the factors (Fig. 3).

#### Accuracy

For accuracy, there was a significant main effect of all three factors. First, there was a main effect of Familiarity, *F*(1, 25) = 274.27, *p* < 0.001, η_p_^2^ = 0.916, with accuracy for familiar faces being higher than for unfamiliar faces (familiar = 87.9%, unfamiliar = 61.5%). There was also a main effect of Template, *F*(1, 25) = 17.45, *p* < 0.001, η_p_^2^ = 0.411, with the multiple image template having higher accuracy than the average template (multiple = 77.3%, average = 72.1%). The main effect of Set Size was also significant, with increasing Set Size causing a decrease in accuracy, *F*(2,50) = 136.34, *p* < 0.001, η_p_^2^ = 0.845 (Set Size 5 = 86.8%, Set Size 10 = 76.2%, Set Size 20 = 61%). There was a significant interaction between the Familiarity and Template factors, *F*(1, 25) = 22.94, *p* < 0.001, η_p_^2^ = 0.478. Simple main effects analysis reveal that multiple image template has significantly higher accuracy than the average template for unfamiliar faces, *F*(1, 25) = 43.03, *p* < 0.001, η_p_^2^ = 0.633, but that there was no difference between Templates for familiar faces, *F*(1, 25) = 0.22, *p* = 0.641, η_p_^2^ = 0.009. A significant interaction was also observed between Familiarity and Set Size, *F*(2, 50) = 8.99, *p* < 0.001, η_p_^2^ = 0.265. Based on Fig. 3, it appears that there is a shallower search slope (i.e. smaller cost of larger set size) for familiar than unfamiliar faces. A formal analysis of search slopes is presented below. The three-way interaction, *F*(2, 54) = 0.56, *p* = 0.58, η_p_^2^ = 0.022, and the interaction between Template and Set Size was not significant, *F*(2, 54) = 1.50, *p* = 0.234, η_p_^2^ = 0.056.

#### Response time

For response time, there was an overall main effect of Familiarity, *F*(1, 25) = 9.77, *p* = 0.004, η_p_^2^ = 0.281, with familiar face search faster than unfamiliar face search (familiar = 3.4 s, unfamiliar = 3.9 s). There was also a main effect of Template, *F*(1, 25) = 31.07, *p* < 0.001, η_p_^2^ = 0.554, with multiple image templates resulting in faster search time than average image template (multiple = 3.4 s, average = 3.9 s). We also found a significant main effect for Set Size, *F*(2, 50) = 206.75, *p* < 0.001, η_p_^2^ = 0.892, with increasing Set Size resulting in longer response time (Set Size 5 = 2.4 s, Set Size 10 = 3.5 s, Set Size 20 = 5 s). We also found no significant three way interaction, *F*(2, 50) = 1.70, *p* = 0.193, η_p_^2^ = 0.064, nor two way interactions between Familiarity and Template, *F*(1, 25) = 2.48, *p* = 0.128, η_p_^2^ = 0.090, Familiarity and Set Size, *F*(2, 50) = 0.24, *p* = 0.784, η_p_^2^ = 0.010, or Template and Set Size, *F*(2, 50) = 1.39, *p* = 0.260, η_p_^2^ = 0.052.

#### Search slopes

To compare the speed and accuracy that distractors are rejected, we also calculated and compared the search slopes for each condition (Tong & Nakayama, 1999; Treisman & Souther, 1985). Search slopes were calculated for both accuracy and response time function and analyzed in a 2 × 2 repeated measures ANOVA with Familiarity and Template as the factors. Slopes were calculated for each participant individually using linear modelling by the least squares method, with the gradient of the model giving the measured search slope.

For accuracy, we found a significant main effect of Familiarity, *F*(1, 25) = 5.5, *p* = 0.007, η_p_^2^ = 0.255, with the slope for familiar faces being significantly shallower than for unfamiliar faces, suggesting that familiarity reduced the detrimental impact a larger search array had on P’s ability to find the target. The main effect of Template, *F*(1, 25) = 0.09, *p* = 0.767, η_p_^2^ = 0.004, and interaction was not significant, *F*(1, 25) = 0.88, *p* = 0.357, η_p_^2^ = 0.034.

For response time, we found no significant main effect of Familiarity, *F*(1, 25) = 0.32, *p* = 0.577, η_p_^2^ = 0.019, or Template, *F*(1, 25) = 2.03, *p* = 0.166, η_p_^2^ = 0.075, nor interaction between these two factors, *F*(1, 25) = 0.59, *p* = 0.450, η_p_^2^ = 0.023, confirming that these factors did not interact with the response time search slope.

### Discussion

In Experiment 3, we have found that search templates elicited from multiple images lead to faster and more accurate search than those from face averages. These results extend the findings of studies of unfamiliar face matching (White et al., 2014), where multiple images have been shown to lead to more accurate matching performance than face averages. Although face averages contain the invariant features of a face, it appears that the variance information contained in multiple images produces more robust templates for visual search.

Moreover, the advantage for multiple images occurred despite substantial differences in the quantity of image information used to derive these representations: multiple image arrays consisted of four images and face averages were generated from 19 images. This discrepancy may mask a larger advantage for multiple images than reported here, as combining more images into the face averages may improve subsequent identifications (Burton et al., 2005). Given that access to images of targets may be limited in real-world tasks, this suggests that face averages are of limited applied use for this task.

Another aim of this experiment was to determine whether the number of distractors affected changes in familiarity and the search template. Our results show that performance declines precipitously with increasing number of distractors. However, for accuracy scores at least, this effect is substantially larger for unfamiliar faces than for familiar faces. Because neither familiarity nor multiple image exposure provide additional benefits to response time as the number of distractors increases, this suggests that these familiarity-based improvements are not a result of faster distractor rejection. Because face processing is capacity-limited, with only one face being processed at a time (Bindemann, Burton, & Jenkins, 2005; Bindemann, Jenkins, & Burton, 2007), these findings support the conclusion that searching for a particular face, whether familiar or unfamiliar, must be performed serially (Nothdurft, 1993; Tong & Nakayama, 1999; Wolfe & Horowitz, 2004).

Overall, the results of Experiment 3 show that becoming familiar with a face can help protect against costly false-positive errors when searching for faces in crowds and that partial benefits of familiarity can be reached by exposing participants to image sets that have naturalistic variation in facial appearance.

## General discussion

These studies are the first to examine the accuracy of visual search performance for faces using images that have natural variations in appearance. When faces were unfamiliar to the viewer, performance was extremely poor. When searching for an unfamiliar target face among 19 distractors, participants chose the wrong face on approximately half of all trials. This is surprisingly poor accuracy, especially considering that the distractor faces were not chosen to be similar to the target face and even included faces of the opposite sex (men as distractors for female targets and vice versa). This suggests that earlier estimates of visual search accuracy for unfamiliar faces may profoundly underestimate the difficulty of this task in real-world settings (Ito & Sakurai, 2014; Tong & Nakayama, 1999).

It is commonplace for surveillance operations to be undertaken in crowded spaces containing far greater numbers of distractor faces than we included in this study. The very clear and detrimental effect of including more distractors in our arrays in Experiment 3 suggests that, in these situations, the probability of false identifications would far outweigh the probability of a correct identification. For example, in Experiment 3, the accuracy fell from 86.8% with a crowd size of five, to as low as 61% with a crowd size of just 20 faces. This raises serious questions about the utility of asking people to search for faces in crowds.

Although search accuracy for unfamiliar faces was concerning, the benefits of familiarity were clear and may provide a partial solution to this problem. Across three experiments, we found that performance is substantially better when searching for a familiar face. For example, in Experiment 3 the benefit of familiarity on search accuracy increased with the number of distractors from 15.8% to 32.8%. This benefit is higher than those found earlier in face matching tasks, where familiarity led to an increase of no more than 20% (White et al., 2014). One of the reasons for this may be that we coded familiarity on a participant-by-participant basis. This approach would have meant that any of the Australian celebrities that were unfamiliar to participants would be marked as such. This is distinct from previous studies that have assumed that all of the local celebrities were familiar to participants (Ritchie et al., 2015; White et al., 2014) and means that these studies may have underestimated the size of the familiarity benefit for face matching (see Additional file 1 for analysis).

A further aim of these studies was to determine whether we could compensate for a lack of familiarity with a face by changing the visual search template. First, we found that face averages improved search by increasing the accuracy that participants selected the target from the search array. Second, we found that multiple images also improved search performance by increasing both the speed and accuracy that participants selected the target. Last, we found that multiple images outperformed face averages, allowing targets to be found both more quickly and accurately. Importantly, these improvements were achieved with very few images and very brief image exposure periods. This suggests that when more images are available, and images can be studied for longer periods, larger benefits may be achieved.

While we were able to improve performance by altering the visual information contained in search templates, these improvements were very small in comparison to the benefits of familiarity. Searching for a familiar face is a more efficient process, allowing familiar faces to be located much faster and more accurately than unfamiliar faces. For example, searching for an unfamiliar face is exhaustive and requires participants to scan each item in the array to confirm the target’s location; however, searching for a familiar face is more often terminated after the target is first encountered (Ito & Sakurai, 2014). This change in search strategy reveals something fundamentally different about searching for a familiar face; that is, we know when to stop looking. This allows familiarity to protect against making false-positive errors and reach a decision more quickly. In applied security settings, this reduction in false identifications might be particularly valuable, leading to a reduction in wasted time and decreasing the chances of innocent people being falsely accused.

Familiar face search can serve as a useful benchmark for the level of accuracy that is attainable on this task. Future work should therefore devise methods that make best use of images for familiarizing viewers with previously unfamiliar faces. Exposing to variation in images of unfamiliar faces has been shown to result in improvements in perceptual matching (e.g. Andrews, Jenkins, Cursiter, & Burton, 2015; Ritchie & Burton, 2017; White et al., 2014), recognition memory (e.g. Etchells et al., 2017; Menon et al., 2015b; Murphy et al., 2015), and name verification (Ritchie & Burton, 2017). For example, it appears possible to accelerate the familiarization process by asking participants to discriminate images of the unfamiliar target from images showing a similar looking individual (Dowsett, Sandford, & Burton, 2016). In addition, by modelling image statistics it may be possible to generate larger sets of variable images where a limited set of images are available (see Burton, Kramer, Ritchie, & Jenkins, 2016). Given that we find substantial benefits following brief exposure to just four images, there appears to be significant scope to amplify these benefits in future work.^2^

Another avenue for future work aiming to improve the accuracy of face-in-the-crowd search is to examine individual differences in performance on this task. While it was not the purpose of the present study to examine individual differences, we did observe large variations in accuracy on this task for all experiments. For example, in Experiment 1, individuals’ accuracy was in the range of 43–73% correct when the average was 58%. Recent research shows stability in these inter-individual differences for face recognition ability, such that people who perform well at one time also perform well when tested at a later date (e.g. Balsdon, Summersby, Kemp, & White, 2018; Wilmer et al., 2010). Assuming that the same competencies for face recognition also drive performance for face search, it may be possible to use challenging tasks such as the one used in the current paper to select high performers to be deployed in critical real-world search tasks. Already, considerable resources are being invested into identifying super-recognizers (Bobak, Pampoulov, & Bate, 2016) who show superior performance on face matching and recognition tasks (Bobak, Dowsett, & Bate, 2016; Bobak, Hancock, & Bate, 2015; Robertson et al., 2016). Because both police and private companies are currently recruiting and deploying super-recognizers for these types of surveillance tasks (see Super-Recognisers International, 2018), it is important to establish whether the individuals with high levels of aptitude in face matching tasks also perform well when faced with the challenging face-in-a-crowd tasks that their professional roles entail (e.g. Davis et al., 2018, see also Valeriani, Cinel, & Poli, 2017).

There are limitations on the applicability of the experiments reported here to real-world crowd search. First, our experiments required participants to locate a target that was always present in the search array. This is unrealistic because in real-world tasks targets may be absent from the crowd. Second, while these studies focused on identifying faces in crowds, in the ambient environments where these searches occur many other cues can be used to facilitate identification. For example, a person’s body can also be a reliable cue for person identification even under poor viewing conditions (O’Toole et al., 2011; Rice, Phillips, & O’Toole, 2013). Therefore, our experiments provide a starting point for understanding how face-in-crowd search operates in unconstrained environments. Future research is necessary to provide a comprehensive picture of the factors that contribute to the person-in-crowd searches that underpin the tasks performed in many important security and surveillance roles.

## Conclusions

We report poor accuracy when searching an unfamiliar face in a “crowd” of images that contain natural day-to-day variation in appearance, with performance markedly lower than in studies using studio-captured images. Familiarity was effective at reducing these errors, resulting in a substantially faster and more accurate search for the target face. Further, we were able to improve performance by presenting participants with image averages or multiple images of an unfamiliar target. Providing multiple images conferred an additional advantage over face averages; therefore, we conclude that exposing participants to variable images of the search target is a promising route to improving accuracy of face-in-crowd search in real-world tasks.

## Additional file

Additional file 1: Familiarity analysis. (DOCX 29 kb)

## Abbreviations

CCTV: Closed-circuit television
SD: Standard deviation units

## Footnotes

1 In all experiments, we assessed familiarity individually for each participant. This approach led to some celebrity faces that were not correctly identified being included in the unfamiliar condition. Although participants were unable to provide the names or semantic information about these celebrities, they may have been visually familiar to participants; therefore, accuracy in the unfamiliar face condition may have benefited from this residual familiarity. To test this, we reanalyzed data from all experiments without reclassifying trials on the basis of the familiarity check. This analysis is presented in Additional file 1 and shows that reanalyzing in this way decreased accuracy in both conditions, suggesting that excluded faces were more familiar than those classified as unfamiliar, but less familiar than those classified as familiar.

2 In future work, it will also be important to examine the mechanisms by which exposure to variability facilitates performance. Indeed, there are competing accounts of the present results that cannot be distinguished on the basis of data presented here. One account of the multiple image advantage is that it increased the likelihood that one image in the search template was a close match to the appearance of the target in the search array. An alternative account is that exposure to variability led to an integrated identity representation (see Etchells et al., 2017; Menon et al., 2015a; Menon et al., 2015b). Distinguishing between the alternate explanations is of practical and theoretical value and warrants further exploration.
