# Supplementary material for: Search templates that incorporate within-face variation improve visual search for faces
Source: Cogn Res Princ Implic. 2018 Sep 26;3:37. doi: 10.1186/s41235-018-0128-1 (PMC6156691; doi:10.1186/s41235-018-0128-1)
Supplement: Supplementary file 1 — Familiarity analysis. (DOCX 29 kb) [file 41235_2018_128_MOESM1_ESM.docx]

**Search templates that incorporate within-face variation improve visual search for faces**

**Additional file 1: Familiarity analysis**

James D. Dunn, Richard I. Kemp & David White

*Corresponding author: j.d.dunn@unsw.edu.au*

In all experiments, we applied a strict criterion for classifying faces as familiar by assessing each participant’s familiarity with the celebrity identities. An alternative approach -- which has been used in previous research -- is to assume participants are familiar with all of the famous celebrities and are unfamiliar with all of the Dutch celebrities in the experiment (Ritchie et al., 2015; White et al., 2014). Therefore, we reanalysed accuracy and response time data for all three experiments using this more liberal approach.

Full details of this analysis are reported below. To summarise, analysing performance data using this more liberal approach led to lower accuracy for both unfamiliar and familiar face conditions compared to when familiarity was classified on a by-item basis using data from the familiarity check (as reported in the main body of the paper). Averaging across experiments, we observed a 7.5% increase in accuracy for familiar faces and 4.3% for unfamiliar faces. Thus, participants appear to have had some level of familiarity with the excluded celebrities, leading to better accuracy for these faces when compared to the faces classified as unfamiliar, but reduced accuracy when compared to faces that are more familiar.

## Experiment 1

We analysed the effect of coding familiarity individually vs. assuming for both accuracy and response time separately using two paired sample t-tests using Bonferroni adjusted alpha levels of .025 per test (.05/2).

### Accuracy

Accuracy was significantly higher for unfamiliar faces when familiarity was coded individually (45.8% vs. 40.1%), *t* (25) = 4.86, *p* < .001, Cohen’s *d* = 0.95. This result shows that requiring participants name the celebrities for them to be categorised as familiar causes higher observed accuracy. One explanation for this is that participants have some visual familiarity that, despite not being able to identify them, is be beneficial for search performance. Accuracy was also higher for familiar faces when familiarity was coded individually (90.4% vs. 76.4%), *t* (25) = 6.75, *p* < .001, Cohen’s *d* = 1.32. Here we see the main benefit of coding familiarity individually, as it causes much higher observed accuracy in the familiar face condition. This is likely due to the removal of less familiar international celebrities that would otherwise lower observed accuracy.

### Response Time

For response time, there was no significant difference between the two different methods for unfamiliar faces (7.8s vs. 7.8s), *t* (25) = -1.09, *p* = .285, Cohen’s *d* = -0.21. However, there was a significant difference between methods for familiar faces, *t* (25) = 6.12, *p* < .001, Cohen’s *d* = 1.20, with faster reaction times observed in the familiar face condition when familiarity was coded individually (5.7s vs. 6.7s). Again, this suggests that removal of less familiar international celebrities increases the observed benefit of familiarity for search speed.

## Experiment 2

Analysis was performed here in the same way as Experiment 1.

### Accuracy

Accuracy was significantly higher for unfamiliar faces when familiarity was coded individually (52.4% vs. 47.9%), *t* (25) = 6.64, *p* < .001, Cohen’s *d* = 1.30. This result again shows how these different methods affect the accuracy observed for unfamiliar faces. Accuracy was also higher for familiar faces when familiarity was coded individually (92.6% vs. 84.6%), *t* (25) = 5.23, *p* < .001, Cohen’s *d* = 1.03. Again, coding familiarity individually increases the observed accuracy for familiar faces.

### Response Time

For response time, surprisingly the observed speed was slower for unfamiliar faces with familiarity coding (6.4s vs. 6.6s), *t* (25) = -3.14, *p* = .004, Cohen’s *d* = -0.61. The observed speed was also significantly faster for familiar faces with individual coding (5.4s vs. 6s), *t* (25) = 4.25, *p* < .001, Cohen’s *d* = 0.83, with faster reaction times observed in the familiar face condition when familiarity was coded individually (5.7s vs. 6.7s). Again, this suggests that removal of less familiar international celebrities increases the observed benefit of familiarity for search speed.

## Experiment 3

We analysed the effect of coding familiarity individually vs. assuming for both accuracy and response time separately using six paired sample t-tests (separately for each set size) using Bonferroni adjusted alpha levels of .008 per test (.05/6).

### Accuracy

Observed accuracy was significantly higher for unfamiliar faces with familiarity coding than without for set size 10 (62.4% vs. 58%) and 20 (45.5% vs. 40.8%) but not 5 (78.6% vs. 76.5%), Set Size 5: *t* (25) = 2.37, *p* = .026, Cohen’s *d* = 0.47; Set Size 10: *t* (25) = 4.54, *p* < .001, Cohen’s *d* = 0.89; Set Size 20: *t* (25) = 4.80, *p* < .001, Cohen’s *d* = 0.94. Similarly, the observed accuracy was also significantly higher for familiar faces with familiarity coding for set size 10 (91.7% vs. 85.6%) and 20 (77.4% vs. 72.1%) but not 5 (95.4% vs. 91.1%), Set Size 5: *t* (25) = 2.67, *p* = .013, Cohen’s *d* = 0.82; Set Size 10: *t* (25) = 3.98, *p* = .001, Cohen’s *d* = 0.78; Set Size 20: *t* (25) = 3.03, *p* = .006, Cohen’s *d* = 0.59. Together these results show how familiarity increases the observed accuracy for both unfamiliar and familiar face conditions in this experiment.

### Response Time

For response time, there were no significant differences for unfamiliar faces at any level of set size 5 (2.6s vs. 2.6s), 10 (3.7s vs. 3.7s) and 20 (5.1s vs. 4.8s) Set Size 5: *t* (25) = -1.48, *p* = .153, Cohen’s *d* = -0.29; Set Size 10: *t* (25) = -0.61, *p* = .548, Cohen’s *d* = -0.12; Set Size 20: *t* (25) = -2.12, *p* < .001, Cohen’s *d* = 0.94. However for familiar faces, responses were significantly faster with familiarity coding for set size 5 (2s vs. 2.3s), but not 10 (3.2s vs 3.3s) or 20 (4.7s vs 4.9s), Set Size 5: *t* (25) = 5.91, *p* < .001, Cohen’s *d* = 1.16; Set Size 10: *t* (25) = 1.37, *p* = .184, Cohen’s *d* = 0.27; Set Size 20: *t* (25) = 2.14, *p* = .042, Cohen’s *d* = 0.42.
